# Modeling the pharmacokinetic‐pharmacodynamic relationship of the monoclonal anti‐macaque‐IL‐15 antibody Hu714MuXHu in cynomolgus monkeys

**DOI:** 10.1002/prp2.199

**Published:** 2016-01-11

**Authors:** Wei. J. Pan, Hong Li, Jim J. Xiao, Michelle J. Horner, Herve N. Lebrec, Eric A. Butz, Arunan Kaliyaperumal, Tsui C. Cheah, Robert C. Ortiz, Samantha P. Prokop, Sabina A. Buntich, Babette M. Boren, Suzanne T. Wolford, Wayne H. Tsuji, Larry C. Wienkers, Kathleen Köck

**Affiliations:** ^1^ Pharmacokinetics and Drug MetabolismAmgen Inc.SeattleWashington; ^2^ Pharmacokinetics and Drug MetabolismAmgen Inc.Thousand OaksWashington; ^3^ Comparative Biology and Safety SciencesAmgen Inc.Thousand OaksCalifornia; ^4^ Comparative Biology and Safety SciencesAmgen Inc.SeattleWashington; ^5^ Inflammation Discovery ResearchAmgen Inc.SeattleWashington; ^6^ Medical SciencesAmgen Inc.Thousand OaksCalifornia; ^7^ Covance Laboratories Inc.MadisonWisconsin; ^8^ Medical SciencesAmgen Inc.SeattleWashington

**Keywords:** Anti‐IL‐15 antibody, anti‐inflammatory, cynomolgus monkeys, Hu714MuXHu, natural killer cells, PK/PD modeling

## Abstract

Hu714MuXHu is a recombinant chimeric murine‐human monoclonal antibody directed against interleukin‐15 (IL‐15), a proinflammatory cytokine associated with memory CD8+ and natural killer (NK) T‐cell activation and implicated in the pathogenesis of inflammatory diseases. A pharmacokinetic‐pharmacodynamic (PK/PD) model was developed to describe the NK cell count reduction in cynomolgus monkeys after treatment with Hu714MuXHu. Cynomolgus monkeys were dosed with Hu714MuXHu in three studies: as a single dose at 0.1 or 1 mg·kg^−1^ i.v.; weekly for 5 weeks at 0, 30, 60, or 150 mg·kg^−1^ i.v. or 150 mg·kg^−1^ s.c.; weekly for 13 weeks at 0, 5, 30, or 150 mg·kg^−1^ s.c. Serum Hu714MuXHu concentration‐time data were analyzed using noncompartmental analysis and the PK/NK cell count relationship was assessed via simultaneous PK/PD modeling. Hu714MuXHu PK was approximately dose‐proportional between 0.1–150 mg·kg^−1^ for i.v. and 5–150 mg·kg^−1^ for s.c. administration with an elimination half‐life of 12.7–18 days. Hu714MuXHu administration resulted in rapid and marked reductions in NK cell counts after the first dose which recovered fully after the serum Hu714MuXHu concentrations approached 0.1 *μ*g·mL^−1^ (assay limit of quantification). PK/PD modeled Hu714MuXHu effects on NK cells had an EC_50_ of 0.09 *μ*g·mL^−1^. In summary, weekly i.v. or s.c. doses with Hu714MuXHu for up to 3 months in cynomolgus monkeys demonstrated linear PK and significant NK cell count reduction, which was described using PK/PD modeling. This approach may be used to guide investigative product dose selections for inflammatory diseases where NK cell count alterations are quantifiable.

**Abbreviations**
AAALAC: Association for Assessment and Accreditation of Laboratory Animal Care International
Ab: antibody
AUC: area under the serum concentration‐time curve
AUMC: area under the first‐moment‐time curve
BSV: between‐subject variability
*C*_0_: serum concentration extrapolated to time zero
CD: cluster of differentiation
CL: clearance in serum
*C*_max_: maximum observed serum concentration
*E*_max_: maximal effect
*F*: bioavailability after s.c. administration
GLP: good laboratory practice
HRP: horseradish peroxidase
IACUC: institutional animal care and use committee
EC_50_: concentration at 50% maximal effect
IFN: interferon
IL: interleukin
i.v.: intravenous(ly)
*K*_a_: absorption rate constant after s.c. administration
*K*_in_: formation rate constant of effect
*K*_out_: elimination rate constant of effect
LLOQ: lower limit of quantification
MRT: mean residence time
NK: natural killer
PD: pharmacodynamic(s)
PK: pharmacokinetic(s)
RSE: relative standard error
s.c.: subcutaneous(ly)
*t*_1/2_: terminal half‐life
TK: toxicokinetics
TNF*α*: tumor necrosis factor *α*
*t*_max_: time to *C*_max_
*V*_ss_: volume of distribution at steady state

## Introduction

Interleukin‐15 (IL‐15) is a potent proinflammatory cytokine structurally similar to IL‐2, and mainly produced by dendritic cells, monocytes, macrophages, epithelial and fibroblastic cells, as well as bone marrow stromal cells in response to innate immune stimuli and type 1 interferons (IFN) (Fehniger and Caligiuri 2001). The IL‐15 signal is transmitted through binding to a heteromeric receptor complex comprised of the IL‐2/15R *β* chain, the common *γ* chain, and the IL‐15 specific IL‐15R *α* chain. IL‐15 acts on multiple immune cells and is required for the development, differentiation, and proliferation of memory CD8+ T cells, natural killer (NK) cells, and NK T cells (Grabstein et al. 1994; Waldmann and Tagaya 1999). Furthermore, IL‐15 has been shown to enhance survival and activation of dendritic cells (Mattei et al. 2001; Gil et al. 2010). IL‐15 acts early in inflammatory disease and induces the production of proinflammatory cytokines such as IFN*γ* and tumor necrosis factor *α* (TNF*α*) through recruitment and activation of inflammatory cells (McInnes et al. 1997; Musso et al. 1998).

Increased levels of IL‐15 in peripheral blood and tissue have been demonstrated in a variety of autoimmune inflammatory disease including rheumatoid arthritis (McInnes et al. 1997; Gonzalez‐Alvaro et al. 2003, 2011), systemic lupus erythematosus (Baranda et al. 2005), psoriasis (D'Auria et al. 1999; Ong et al. 2002), and celiac disease (Maiuri et al. 2000; De Nitto et al. 2009; Meresse et al. 2012; Abadie and Jabri 2014), as well as graft‐versus‐host disease, and solid organ transplant rejection (Smith et al. 2002), suggesting that IL‐15 plays a critical role in the pathogenesis of these diseases or conditions. It has been hypothesized that inhibition of IL‐15 may reduce inflammatory response and treat these various autoimmune diseases or inflammatory conditions. Several agents that inhibit IL‐15 action have been described in the literature. Neutralization of IL‐15 using the soluble IL‐15 receptor *α* or blockage of the IL‐15 receptor ameliorated murine collagen‐induced arthritis, and antibodies directed against IL‐15 have been shown to be effective in a mouse model with human psoriasis xenografts (Ruchatz et al. 1998; Villadsen et al. 2003; Ferrari‐Lacraz et al. 2004). In addition, inhibition of IL‐15 in a mouse model of celiac disease, induced apoptosis of intraepithelial lymphocytes, reduced their accumulation in the gut epithelium, and was able to block the antiapoptotic cascade in intraepithelial lymphocytes and proinflammatory signaling in biopsies of human celiac disease (Benahmed et al. 2007; Malamut et al. 2010).

Data in preclinical species have highlighted the important role of IL‐15 in the development and survival of NK cells. IL‐15 as well as IL‐15R*α* knockout mice have reduced levels of NK cells (Lodolce et al. 1998; Kennedy et al. 2000), whereas exogenous administration of IL‐15 could restore NK cell counts in IL‐15 knockout mice (Kennedy et al. 2000). In cynomolgus monkeys, IL‐15 receptor blockage resulted in reduced numbers of NK cells in peripheral blood (Haustein et al. 2010). Similarly, tofacitinib (CP‐690,550), a Janus kinase 3 (JAK3) inhibitor that blocks signaling through the common *γ* chain of cytokine receptors including those for IL‐15, has been shown to reduce NK cell counts in cynomolgus monkeys (Conklyn et al. 2004). However, while IL‐15 may be important to the development of human NK cells (Grabstein et al. 1994; Waldmann and Tagaya 1999), it does not appear to be required for human NK cell homeostasis (Lebrec et al. 2013).

We have developed a human immunoglobulin G_1_ (IgG_1_ *κ*) monoclonal antibody AMG 714 which specifically binds to and inhibits the functions of human IL‐15. In vitro studies demonstrated that AMG 714 had high‐binding affinity for human IL‐15, but lower affinity for macaque IL‐15. Additionally, AMG 714 neutralized human IL‐15 but did not efficiently neutralize macaque IL‐15. To enable preclinical studies in macaques, a surrogate antibody, Hu714MuXHu, was developed by fusing the antibody‐binding portion [F(ab)] of a mouse anti‐human IL‐15 monoclonal antibody known to neutralize macaque IL‐15, M111, with a human IgG_1_ constant region (Fc). Hu714MuXHu was shown to neutralize macaque IL‐15 with approximately the same potency as AMG 714 neutralizes human IL‐15 in vitro.

The objectives of our investigations were to determine the pharmacokinetics (PK), to explore pharmacodynamics (PD), and to model the PK and effects on NK cell count after single or multiple Hu714MuXHu intravenous (i.v.) or subcutaneous (s.c.) administration in male and female cynomolgus monkeys. To our knowledge, this is the first report on the PK/PD modeling of an anti‐IL‐15 antibody. However, because the reduction in NK cell counts in cynomolgus monkeys was not observed in healthy humans after administration of AMG 714, the utility of our PK/PD model might be restricted to certain clinical disease conditions. For example, although survival of human NK cells is IL‐15 independent (Lebrec et al. 2013), a recent study in cancer patients demonstrated influx and hyperproliferation of NK cells in the peripheral blood after IL‐15 administration (Conlon et al. 2015). These data suggest that IL‐15 blockade might only affect NK cell counts under conditions where IL‐15 levels are elevated (e.g., in patients with inflammatory diseases). In addition, the presented PK/PD modeling approach may be useful for guiding therapeutic dose selection of investigational agents that target the IL‐15 pathway such as JAK3 inhibitors (Conklyn et al. 2004; Borie et al. 2005).

## Materials and Methods

### Test material

Hu714MuXHu (molecular weight: 144 kDa) is a chimeric monoclonal antibody with a murine variable region grafted on a human IgG_1_ constant region. It is a surrogate for the fully human monoclonal antibody AMG 714, which is directed against human IL‐15. Hu714MuXHu binds to cynomolgus monkey and human IL‐15 with comparable affinity, with *K*_D_ values of 120 ± 60 pmol*L^‐1^ and 240 ± 40 pmol*L^‐1^, respectively. Hu714MuXHu and AMG 714 bind to overlapping epitopes on human IL‐15 and do not interfere with IL‐15 binding to IL‐15 receptor *α*. Instead, they prevent IL‐15 signaling by blocking assembly of the complete receptor complex. However, whereas AMG 714 does not efficiently neutralize cynomolgus monkey IL‐15, Hu714MuXHu does, and has thus been used for preclinical testing in cynomolgus monkeys.

The investigational product was formulated with pharmaceutically accepted excipients at a nominal concentration of 30 mg**·**mL^−1^ and stored at −60 to −80°C until use. At least 24 h prior to dose preparation, the test article was transferred to a refrigerator at 2–8°C. The control article was formulated with the same excipients and stored at 2–8°C.

### Animal husbandry

Naïve male (*N* = 55) and female (*N* = 49) cynomolgus monkeys (*Macaca fascicularis*) of Chinese origin (*N* = 104 total, age: 3–6 years, weight: 2.0–4.6 kg) were obtained for the conduct of the studies. Experiments were performed in facilities accredited by the Association for Assessment and Accreditation of Laboratory Animal Care International (AAALAC; Frederick, MD). The experiments were conducted in compliance with the most recent version of the United States Food and Drug Administration (US FDA) Good Laboratory Practice Regulations, 21 CFR part 58; the Japanese Ministry of Health, Labor, and Welfare (MHLW), Good Laboratory Practice (GLP) Standards, Ordinance 21; the Organization for Economic Cooperation and Development (OECD) Principles of GLP, C(97) 186/Final; and with any applicable amendments. This research also adhered to the “Principles of Laboratory Animal Care” (US National Institute of Health Publication #85‐23, revised in 1985). Results obtained from animal studies have been reported in accordance with the ARRIVE guidance (Kilkenny et al. 2010).

The monkeys were acclimated to laboratory conditions for at least 4 weeks and housed individually in stainless‐steel cages, except when comingled for environmental enrichment unless precluded for behavior or health reasons. The environment was maintained within a temperature range 18–29°C, relative humidity range 30–70%, 10 or greater air changes/hour and a 12‐h light/dark cycle. Unless fasted, certified primate diet was available ad libitum two times daily; purified tap water was available ad libitum. Fruits, vegetables, and toys were given as a form of environmental enrichment.

### Dose administration

Serum Hu714MuXHu concentration‐time and pharmacodynamic data for the current manuscript were obtained from three studies in cynomolgus monkeys: a single‐dose PK and PD study and 1‐month (5 weekly doses) and 3‐month (13 weekly doses) GLP toxicology studies. The test article was administered to cynomolgus monkeys based on body weight as a single‐dose i.v. administration into the saphenous vein, or once‐weekly for 1 or 3 months via i.v. or s.c. in a saphenous vein or the dorsal thoracic region, respectively.

### PK, PD, and immunogenicity assessments

Pre‐ and postdose blood samples for PK and PD measurements were collected as summarized in Table 1. Animals were not fasted for sample collections (unless the collection was concurrent with clinical pathology sampling).

**Table 1:** Hu714MuXHu treatments and procedures for studies in cynomolgus monkeys

Study	*N*	Groups	Treatment	Blood sample collection for PK	Blood sample collection for PD^a^
Single dose	6 males	2 groups; 3 per group	0.1 mg·kg^−1^ i.v. 1 mg·kg^−1^ i.v. Single dose	Predose, and on Days 3, 5, 8, 14, 21, 28, 35, and 42	2 times at baseline; predose, and on Days 3, 5, 8, 14, 21, 28, 35, and 42
1‐month GLP	25 males 25 females	5 groups; 5 per sex per group	Vehicle control i.v. 30 mg·kg^−1^ i.v. 60 mg·kg^−1^ i.v. 150 mg·kg^−1^ i.v. 150 mg·kg^−1^ s.c. QW for 4 weeks	Predose, and 0.5, 2, 8, 24, 96, and 168 h postdose on Days 1 and 22, and predose on Day 15. In recovery monkeys (2/sex per group), on Days 43, 57, 71, 85, 113, 141, 169, 197, 225, 253, 281, 309, and 337	2 times at baseline, predose on Day 15, and on Day 30. In recovery monkeys, on Days 57, 85, 113, 141, 169, 197, 225, 253, 281, 309, and 337
3‐month GLP	24 males 24 females	4 groups; 6 per sex per group	Vehicle control s.c. 5 mg·kg^−1^ s.c. 30 mg·kg^−1^ s.c. 150 mg·kg^−1^ s.c. QW for 13 weeks	Predose, and 0.5, 2, 8, 24, 48, and 168 h postdose on Day 1. Predose, and 0.5, 2, 8, 24, 96, and 168 h postdose on Day 85. Predose on Days 29 and 57 and 1 day after day 92 dose. In recovery monkeys (2/sex/group), on Days 106, 120, 134, 148, 176, and 205	3 times at baseline; predose 1 and on Day 3; predose on Days 8, 15, 29, and 57 and on Day 93. In recovery monkeys, on Days 120, 148, 176, and 205

i.v., intravenously; s.c., subcutaneously; PD, pharmacodynamics; PK, pharmacokinetics; QW, once‐weekly.

a PD assessment via immunophenotyping.

For the determination of serum Hu714MuXHu concentrations, blood samples were collected via the femoral vein into plain tubes with no anticoagulant. Blood samples were allowed to clot for at least 1 h (but not longer than 4 h); after centrifugation at 2000*g* for 10 min, serum was aliquoted, stored at −60 to −80°C and subsequently shipped to Amgen Inc. for analysis. Serum samples from the three studies were analyzed for Hu714MuXHu using a validated quantitative enzyme‐linked immunosorbent assay (ELISA) with a lower limit of quantification (LLOQ) of 0.1 *μ*g**·**mL^−1^. Briefly, microplate wells were coated with monoclonal anti‐Hu714MuXHu antibody to capture Hu714MuXHu. Standard and quality control samples, made by spiking Hu714MuXHu into 100% cynomolgus monkey serum, as well as blanks and study samples were loaded into the microplate wells after a dilution of 1:100 with buffer. Following incubation, unbound material was removed by washing. The detection reagent, biotinylated polyclonal anti‐Hu714MuXHu, was then added to the wells. After removal of the unbound biotinylated antibody by washing, streptavidin conjugated poly‐horseradish peroxidase (HRP; Thermo, IL) was added to each well. After another washing step, a tetramethylbenzidine solution (Bio FX Laboratories, MD) was added to the wells. After stopping the color development, the optical density was measured at 450–650 nm. Data were reduced using the Watson version 7.0.0.01 data reduction package using a 5‐parameter (auto estimate) regression model.

For PD assessments, blood samples were collected from a femoral vein into potassium EDTA tubes. NK cells were analyzed as part of immunophenotyping using the following clusters of differentiation (CDs): total T cells (CD3+), helper T cells (CD3+CD4+), naive helper T cells (CD3+CD8‐CD45RA+), memory helper T cells (CD3+CD8‐CD45RA‐), cytotoxic T cells (CD3+CD8+), naive cytotoxic T cells (CD3+CD8+CD45RA+), memory cytotoxic T cells (CD3+CD8+CD45RA‐), B cells (CD3‐CD20+), NK cells (CD3‐CD16+).

Serum samples from the monkey studies were assessed for anti‐Hu714MuXHu binding antibodies using a validated acid dissociation bridging ELISA. Briefly, Hu714MuXHu was covalently attached to the wells of a 96‐well microtiter plate via amine coupling. The wells were then blocked with milk diluents. Serum samples were diluted in acetic acid and left at room temperature for antibody complex dissociation. A biotinylated Hu714MuXHu secondary reagent was prepared and added to each well of the plate. TRIS was then added to each well just prior to the addition of the acid‐treated serum samples. The serum samples, at neutral pH, were mixed and left at room temperatures to allow anti‐Hu714MuXHu antibodies to bind to the covalently attached Hu714MuXHu. The plate was washed with wash buffer and peroxidase‐conjugated streptavidin was added to each well. The plate was washed again and a tetramethylbenzidine substrate solution was added for color development. The enzymatic reaction was stopped with phosphoric acid and the plate was read on a calibrated spectrophotometer to determine the optical density of each well. The LLOQ was 0.25 *μ*g**·**mL^−1^ in the presence of 10 *μ*g**·**mL^−1^ of Hu714MuXHu in cynomolgus serum.

### PK analysis and PK/PD modeling

Noncompartmental analyses were performed using WinNonlin^®^ Version 4.1e (Pharsight^®^, a Certara^®^ Company, Sunnyvale, CA) to determine the following PK parameters after single or multiple i.v. or s.c. administrations: observed maximum concentration in serum after s.c. (*C*_max_) and extrapolated to time zero after i.v. (*C*_0_) administration, time to *C*_max_ (*t*_max_), area under the serum Hu714MuXHu concentration‐time curve calculated using the linear/log trapezoidal rule within the dosing interval (AUC_*τ*_, for multiple dosing) or extrapolated to infinity (AUC_∞_, for single dosing), and total body clearance (CL for single i.v. dosing). The rate constant for the terminal log‐linear phase of the concentration‐time curve (*λ*_z_) was estimated using linear regression. The terminal phase elimination half‐life (*t*_1/2_) was calculated as ln2/*λ*_z_. For the single dose study, volume of distribution at steady state (*V*_ss_) was calculated as *V*_ss_ = CL × MRT with MRT being the mean residence time calculated as MRT = AUMC_*∞*_/AUC_*∞*_, where AUMC_*∞*_ is the area under the first‐moment‐time curve extrapolated to infinity.

**Figure 1:**
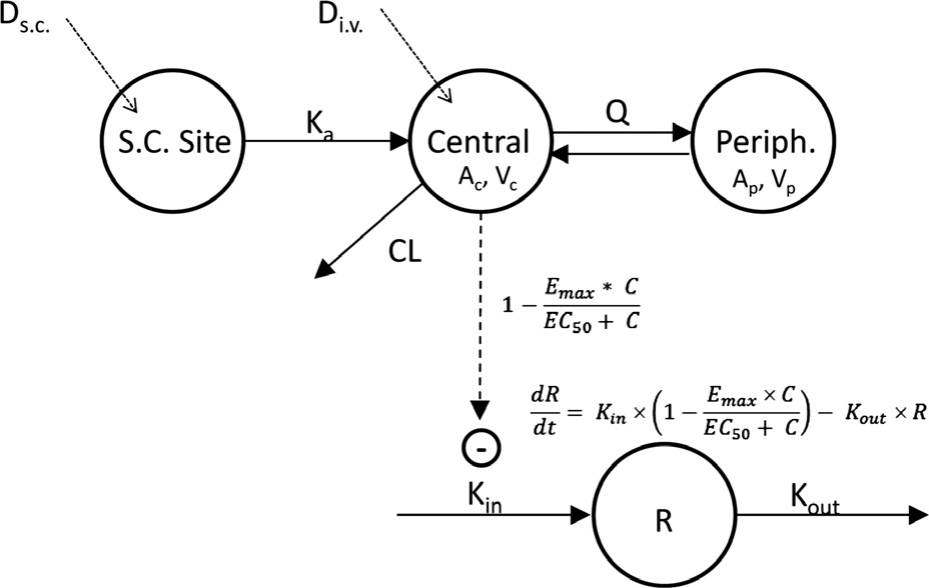
Simultaneous PK/PD modeling of Hu714MuXHu in cynomolgus monkeys: *D*_s.c._ and *D*_i.v._ are subcutaneous and intravenous doses, respectively; *K*_a_ is the absorption rate constant after s.c. administration; CL is the linear elimination clearance from the central compartment; *Q* is the inter‐compartmental clearance between the central and peripheral compartments; *A*_c_, *V*_c_ and *A*_p_, *V*_p_ are central and peripheral amount and volume of distribution for Hu714MuXHu. Hu714MuXHu concentration in the central compartment *C* = *A*_c_/*V*_c_. *R* is the number of NK cells × 10^3^·*μ*L^−1^ of blood with a baseline value of *R*_0_. NK cell turnover rate dR/dt is described by production (*K*_in_) and loss (*K*_out_) rate constants with Hu714MuXHu concentration in the central compartment inhibiting NK cell production. *E*_max_ is the maximal effect, EC_50_ is the concentration producing 50% of the maximal effect. Pheriph, peripheral compartment.

Simultaneous PK/PD modeling was performed using NONMEM^®^ software version VII (ICON Development Solutions, Ellicott City, MD) with the gfortran FORTRAN compiler. The PK/PD model was used to fit the data to characterize the PK properties and the PK‐PD relationship of Hu714MuXHu in cynomolgus monkeys. The structure of the final PK/PD model is shown in Figure 1. The PK was characterized by a two‐compartment model with linear elimination from the central compartment. The PD effect of Hu714MuXHu on NK cells was described by an indirect response model:

$$\frac{dR}{dt}=K_{\mathrm{in}}\times \left(1-\frac{E_{\max}\times C}{EC_{50}+C}\right)-K_{\mathrm{out}}\times R$$

where *R* is the number of NK cells × 10^3^·*μ*L^−1^ in blood with a baseline value of *R*_0_, *C* is the Hu714MuXHu concentration in the central compartment, *E*_max_ is the maximal effect, EC_50_ is the concentration producing 50% of the maximal effect, and *K*_in_ and *K*_out_ are the formation and elimination rate constants of NK cells, respectively. Based on the biology, the effect of Hu714MuXHu concentration on the NK cell population was assumed to be the inhibition of the formation (*K*_in_) of the NK cells.

Data from all three studies were fitted simultaneously with the NONMEM^®^ ADVAN13 subroutine using the first‐order conditional estimation method with INTERACTION (FOCEI) followed by Monte Carlo Importance Sampling (IMP) method and covariance step with MATRIX = S to estimate population PK and PD parameters and to obtain standard error estimates for each parameter. Because log‐normal distribution was assumed for between‐subject variability (BSV), exponential models were used to describe the BSV in the model parameter estimates: *P*_*i*_ = TVP·exp(*η*), where *P*_*i*_ is the individual model parameter for the *i*th subject, TVP is the typical value of the parameter value P, and *η* is a normally distributed random variable with mean of zero and an unknown variance *ω* ^2^ to be estimated. Correlation between BSV of CL and *V* was also considered in the model. The residual variability was first modeled assuming an additive and proportional error model, *Y* = *F* × (1 + *ε*_1_) + *ε*_2_, where *Y* is the PK or PD observation, *F* represents the model prediction values and *ε*_1_ and *ε*_2_ are normally distributed random variables with mean of zero and unknown variance. The additive error *ε*_2_ was fixed to zero in the final model due to its value being consistently estimated to be near zero during model development.

Graphical analyses were performed using R version 3.0.0 for Windows (R project, http://www.r-project.org/) or SigmaPlot version 12.5 (Systat Software, San Jose, CA). Model selection was guided by visual inspection of standard goodness‐of‐fit plots, parameter estimate precision/plausibility, visual predictive check (VPC), and shrinkage of empirical Bayes estimates (Brendel et al. 2007).

## Results

### Pharmacokinetics and immunogenicity response

**Figure 2:**
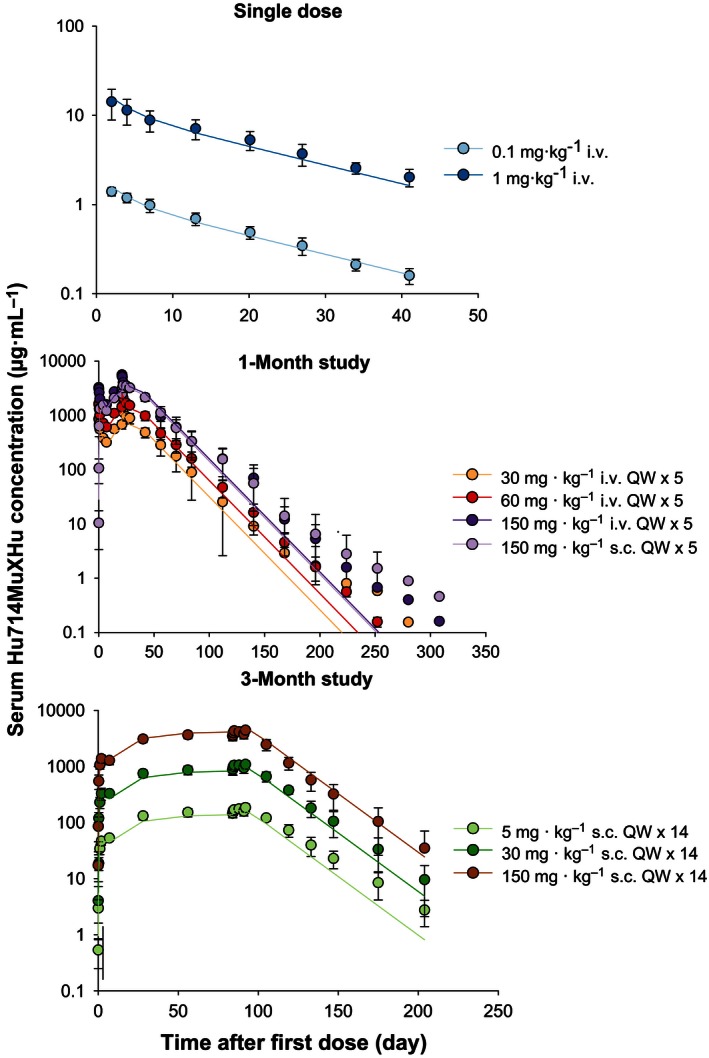
Observed (symbols; mean ± SD) and population predicted (lines) serum Hu714MuXHu concentration‐time profiles after single and multiple i.v. or s.c. administration in cynomolgus monkeys.

Weekly i.v. or s.c. dosing with Hu714MuXHu for up to 3 months in cynomolgus monkeys was well tolerated (data on file at Amgen Inc.). Figure 2 shows the by‐study and by‐cohort observed mean (±SD) and population predicted serum Hu714MuXHu concentration versus time profiles after single or multiple i.v. or s.c. dosing. The curvature for the observed data (symbols) around Day 300 was due to data from less animals (*N* = 2/cohort), which did not follow the preceding general data trend of a typical linear two‐compartment model. Note that, on an individual basis, those animals had PK profiles that did not display curvature around Day 300 (data on file at Amgen Inc.). Table 2 summarizes the estimated noncompartmental Hu714MuXHu PK parameters. Across the studied dose range (0.1–150 mg**·**kg^−1^), Hu714MuXHu exhibited linear kinetics; the exposure parameters *C*_max_ and AUC_*τ*_ of Hu714MuXHu increased approximately dose‐proportionally both when administered as i.v. and as s.c. injections. Based on Day 1 AUC_*τ*_ values, a relative bioavailability of 65% and 75% was calculated for the 30 and 150 mg**·**kg^−1^ s.c. dose cohorts, respectively, compared to those of the corresponding i.v. dose cohorts. In all three studies, no anti‐Hu714MuXHu antibodies were detected in any of the serum samples.

**Table 2:** Mean (SD) Hu714MuXHu noncompartmental pharmacokinetic parameters

Single dose	*N* = 4/Group	0.1 mg·kg^−1^ i.v.	1 mg·kg^−1^ i.v.		
	*C*_0_ (*μ*g·mL^−1^)	1.65 (0.16)	17.9 (7.76)		
	AUC_*∞*_ (*μ*g·day·mL^−1^)	27.2 (4.20)	293 (76.1)		
	CL (mL·day^−1^·kg^−1^)	3.73 (0.56)	3.54 (0.80)		
	*V*_ss_ (mL·kg^−1^)	67.9 (9.6)	77.2 (22.8)		
	*t*_1/2_ (day)	12.7 (0.57)	15.3 (1.3)		

1‐month GLP	*N* = 10/Group	30 mg·kg^−1^ QW i.v.	60 mg·kg^−1^ QW i.v.	150 mg·kg^−1^ QW i.v.	150 mg·kg^−1^ QW s.c.
Day 1	*C*_0_ or *C*_max_ (*μ*g·mL^−1^)	878 (103)	1700 (94.6)	3350 (346)	1560 (203)
	AUC_*τ*_ (*μ*g·day·mL^−1^)	3130 (368)	5850 (344)	12200 (1070)	9100 (1280)
	*C*_0_ or *C*_max_ (*μ*g·mL^−1^)	1490 (172)	2760 (380)	5740 (468)	3640 (489)
Day 22	AUC_*τ*_ (*μ*g·day·mL^−1^)	7210 (1080)	12,900 (1280)	25,300 (2780)	23,900 (3300)
	*t*_1/2_ (day)	15 (4)	16 (1)	17 (1)	16 (4)

3‐month GLP	*N* = 12/group	5 mg·kg^−1^ QW s.c.	30 mg·kg^−1^ QW s.c.	150 mg·kg^−1^ QW s.c.	
Day 1	*C*_max_ (*μ*g·mL^−1^)	53.5 (4.8)	355 (60)	1400 (212)	
	AUC_*τ*_ (*μ*g·day·mL^−1^)	310 (36)	2050 (343)	8450 (1260)	
	*C*_max_ (*μ*g·mL^−1^)	176 (25)	1090 (172)	4370 (545)	
Day 85	AUC_*τ*_ (*μ*g·day·mL^−1^)	1180 (169)	7130 (1170)	28,500 (3880)	
	*t*_1/2_ (day)	18 (2)	16 (2)	16 (4)	

i.v., intravenously; s.c., subcutaneously; QW, once‐weekly; *C*_max_: maximum observed concentration for s.c.; *C*_0_, extrapolated concentration at time zero for i.v.; AUC_*τ*_, area under the concentration‐time curve within the 7‐day dosing interval on Days 1, 22, or 85; AUC_*∞*_, area under the concentration‐time curve from time zero extrapolated to infinity; CL, clearance in serum after i.v. administration; *t*_1/2_, elimination half‐life; *V*_ss_, volume of distribution at steady state after i.v. administration.

Mean *t*_1/2_ values ranged from 12.7 to 18 days and appeared to be dose‐independent. No differences in the PK profiles or PK parameters were observed between female and male monkeys (data on file at Amgen Inc.). In the 3‐month study, the overall Hu714MuXHu accumulation ratio was between 3.4‐ and 3.8‐fold after 13 weeks of repeated weekly dosing. A lower accumulation ratio of 2.1–2.6 was observed in the 1‐month study after 5 weeks of repeated weekly dosing. This discrepancy suggests that steady state was not reached after five doses of Hu714MuXHu, which is consistent with the long half‐life of 12.7–18 days for Hu714MuXHu in these studies (5 half‐lives or 9–13 weekly doses are needed to achieve PK steady state).

### Pharmacodynamics

**Figure 3:**
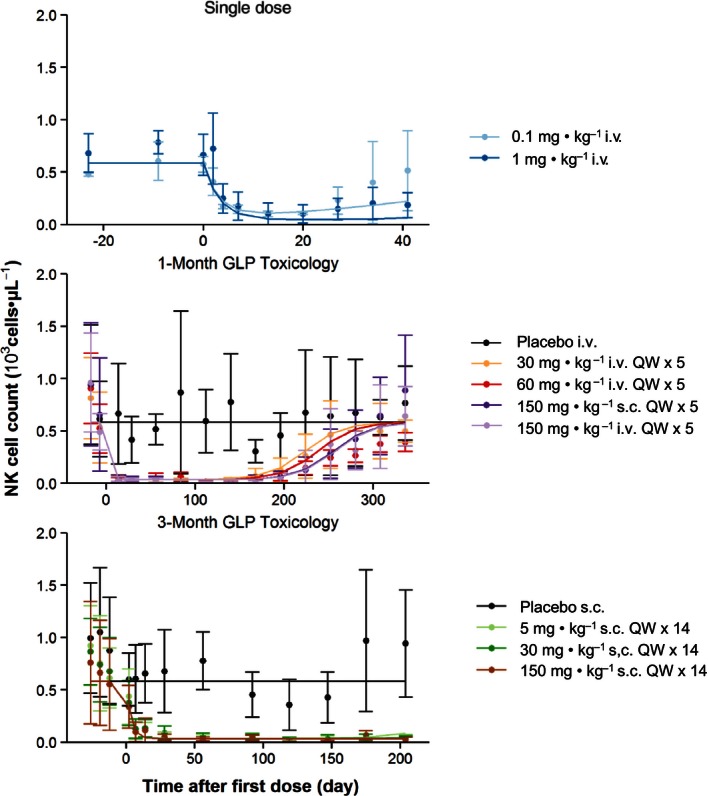
Observed (symbols; mean ± SD) and population predicted (lines) NK cell count‐time profiles after single and multiple i.v. or s.c. administration of Hu714MuXHu in cynomolgus monkeys.

Hu714MuXHu is an antibody that blocks IL‐15 signaling, which is required for development, proliferation, and survival of NK cells. The effect of Hu714MuXHu on NK cell counts are presented as mean (±SD) profiles in Figure 3. Administration of Hu714MuXHu to cynomolgus monkeys resulted in a rapid decline in NK cell counts. NK cell counts returned to predose baseline at 41 days after single 0.1 mg**·**kg^−1^ i.v. dosing; only partial return to baseline at 336 days after the first dose for all 1‐month study active dose cohorts was observed. Due to the limited durations of the follow‐up period, the NK cell count reduction did not reach predose baseline 41 days after single 1 mg**·**kg^−1^ i.v. dose or 204 days after the first dose for any of the 3‐month study active dose cohorts.

### Population PK/PD modeling

A total of 1408 Hu714MuXHu concentration versus time data points and 982 PD (NK cell count) versus time data points from 104 monkeys were included in the simultaneous PK/PD modeling analyses. The final population parameter estimates and between‐subject variability (BSV) of the PK/PD model are presented in Table 3. The population typical values for clearance (CL) and central volume of distribution (*V*_c_) were 3.66 mL**·**day^−1^ **·**kg^−1^ and 44.8 mL**·**kg^−1^, respectively, consistent with what was expected due to the high‐molecular weight of Hu714MuXHu.

**Table 3:** Population PK/PD model parameter estimates for Hu714MuXHu

PK/PD parameters	Parameter estimates (%RSE^a^)	BSV^b^ (%RSE^a^)
*K*_a_ (day^−1^)	0.522 (7.11)	23.7 (38.6)
CL (mL·day^−1^·kg^−1^)	3.66 (3.22)	17.1 (19.8)
*V*_c_ (mL·kg^−1^)	44.8 (4.51)	15.2 (35.7)
*Q* (mL·day^−1^·kg^−1^)	5.07 (17.9)	72.1 (52.9)
*V*_p_ (mL·kg^−1^)	24.3 (8.25)	12.6 (126)
F1	0.824 (3.65)	FIXED
*R*_0_ (10^3^ cells·*μ*L^−1^)	0.584 (4.96)	40.4 (21.7)
*K*_out_ (day^−1^)	0.305 (14.9)	63.2 (34.9)
*E*_max_ (10^3^ cells·*μ*L^−1^)	0.941 (0.386)	2.45 (34.8)
EC_50_ (*μ*g·mL^−1^)	0.0978 (28.0)	90.7 (57.7)
*σ*_1_^2^ (prop. PK)	0.0349	
*σ*_2_^2^ (add. PK)	0 FIXED	
*σ*_3_^2^ (prop. PD)	0.217	
*σ*_4_^2^ (add. PD)	0 FIXED	

See Figure 1 for parameter definitions. *σ* ^12^ − *σ*_4_^2^ are within subject (residual) variance for proportional PK, additive PK, proportional PD, and additive PD errors. All values were rounded to three significant figures.

a Percent standard error of the parameter estimate = standard error of the estimate/parameter estimate × 100.

b Between‐subject variability.

**Figure 4:**
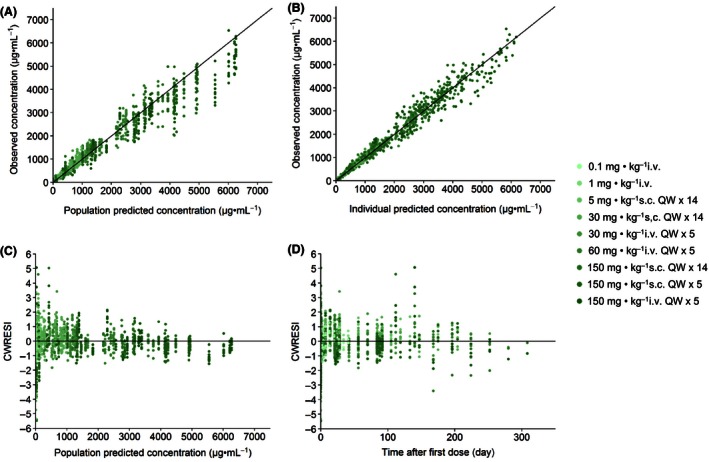
Goodness‐of‐fit plots for Hu714MuXHu concentrations: the observed Hu714MuXHu concentrations were plotted against the population predicted concentrations (A) and the individual predicted concentrations (B). The calculated conditional weighted residuals with interaction (CWRESI) were plotted against the population predicted concentrations (C) and time after first dose (D).

**Figure 5:**
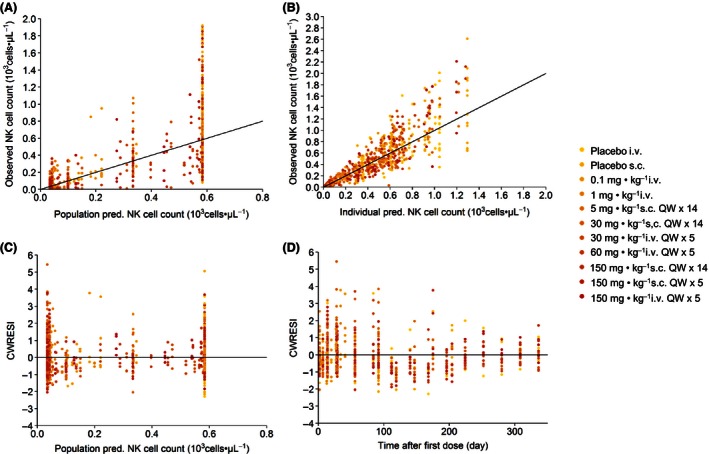
Goodness‐of‐fit plots for NK cell counts after administration of Hu714MuXHu: the observed NK cell counts were plotted against the population predicted NK cell counts (A) and the individual predicted NK cell counts (B). The calculated conditional weighted residuals with interaction (CWRESI) were plotted against the population predicted NK cell counts (C) and time after first dose (D).

**Figure 6:**
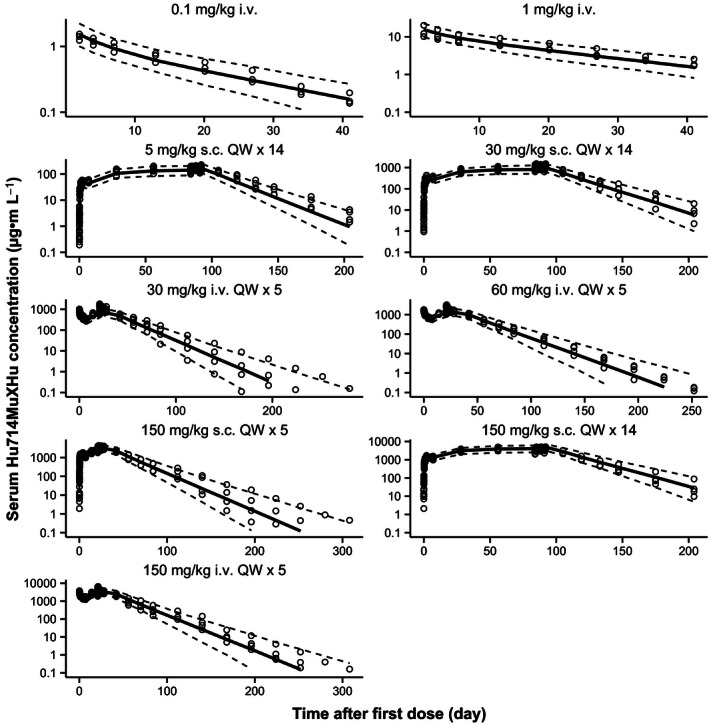
Visual predictive check of PK after single and multiple i.v. or s.c. administration in cynomolgus monkeys. Symbols are individual observed while solid and dashed lines represent the median and the 90% prediction interval, respectively.

**Figure 7:**
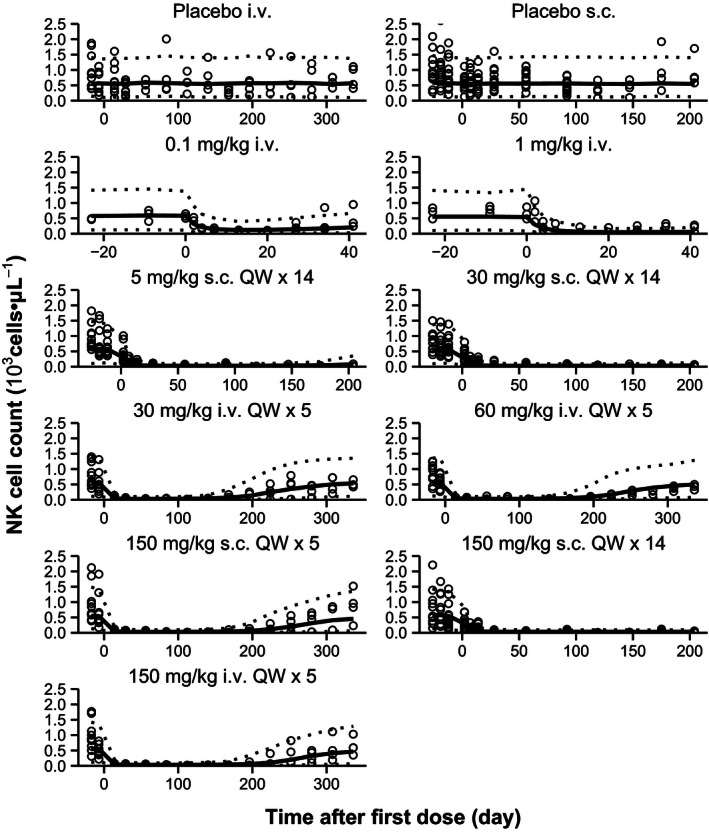
Visual predictive check of NK cell count after single and multiple i.v. or s.c. administration in cynomolgus monkeys. Symbols are individual observed while solid and dashed lines represent the median and the 90% prediction interval, respectively.

The first‐order loss of response (*K*_out_) was estimated to be 0.305 day^−1^ translating into a half‐life of 2.27 days. The estimated values for *E*_max_, EC_50_, and the baseline *R*_0_ were 0.941 × 10^3^ cells**·** *μ*L^−1^, 0.0978 *μ*g**·**mL^−1^, and 0.584 × 10^3^ cells**·** *μ*L^−1^, respectively. For all model‐estimated PK and PD parameters, the relative standard errors (%RSE) were below 30% suggesting that these parameters were estimated with good precision. As Figures 2 and 3 demonstrate, the structural two‐compartment PK/PD model with linear elimination from the central compartment and the *E*_max_ indirect response model adequately describe the serum concentration‐time profiles of Hu714MuXHu and its effects on NK cell counts for all tested dose levels. Based on the goodness‐of‐fit plots, there was a good agreement between the predicted and observed individual data, and no systematic bias was identified in the diagnostic plots of the final model (Figs. 4, 5). Results of the VPC with 500 datasets simulated based on the final PK/PD model parameter estimates are shown in Figures 6 and 7. These plots again demonstrated that the estimated medians and the two‐sided 90% confidence intervals (5th, 50th, and 95th percentiles) of the simulated Hu714MuXHu concentration versus time and NK cell count versus time profiles contain the majority of the individual PK and PD observations.

## Discussion and Conclusions

Interleukin‐15 is an important proinflammatory cytokine that is implicated in the pathogenesis of autoimmune diseases including rheumatoid arthritis (McInnes et al. 1997; Gonzalez‐Alvaro et al. 2003, 2011), systemic lupus erythematosus (Baranda et al. 2005), psoriasis (D'Auria et al. 1999; Ong et al. 2002), and celiac disease (Maiuri et al. 2000; De Nitto et al. 2009). Because multiple immune cells such as CD8+ T cells and NK cells depend on IL‐15 for development, differentiation, and proliferation, blockade of IL‐15 activity has been suggested as a possible treatment strategy for several of the aforementioned autoimmune diseases. We have evaluated the PK and PD of Hu714MuXHu, a recombinant chimeric murine‐human antibody that specifically binds to macaque IL‐15, and developed a PK/PD model to describe the relationship between serum Hu714MuXHu concentrations and reduction in NK cell counts after single i.v. or multiple i.v. or s.c. administrations to cynomolgus monkeys.

Hu714MuXHu PK in cynomolgus monkeys after a single dose i.v. or after weekly i.v. or s.c. doses for up to 3 months was linear within the investigated dose range of 0.1 to 150 mg**·**kg^−1^. The similar mean terminal elimination half‐life (*t*_1/2_) of 12.7–18 days across the investigated doses and duration, as well as two different routes of administration, suggest that the elimination characteristics of Hu714MuXHu were dose‐, time‐, and administration route‐independent. The observed accumulation of Hu714MuXHu in the 1‐ and 3‐month studies is consistent with the long half‐life of this monoclonal antibody. No immunogenicity response (anti‐Hu714MuXHu antibodies) or sex differences were observed.

Consistent with results from previously published studies using tofacitinib (CP‐690550), a JAK3 inhibitor that inhibits signal transduction of the common *γ* chain of cytokine receptors, including IL‐15 (Conklyn et al. 2004; Borie et al. 2005), administration of Hu714MuXHu reduced NK cell numbers in cynomolgus monkeys.

Simultaneous PK/PD modeling using all individual animal data of three studies and all dosing regimens was adequate, with predicted PK parameters matching well with those obtained using noncompartmental analysis. The model‐estimated population values for clearance (CL) and central volume of distribution (*V*_c_) were 3.66 mL**·**day^−1^ **·**kg^−1^ and 44.8 mL**·**kg^−1^, respectively, indicating slow elimination and limited tissue penetration and predominant distribution in serum (central compartment) as expected for this high‐molecular weight protein (Ng et al. 2006; Dirks and Meibohm 2010; Royer et al. 2010).

The PD activity of Hu714MuXHu measured as NK cell count reduction following single or multiple doses was adequately described by the PK/PD model. The rapid decrease in NK cell numbers after Hu714MuXHu administration is consistent with reports in mice, where NK cells transferred into mice with IL‐15 deficiency were lost rapidly. This suggests that macaque NK cell survival is also dependent on IL‐15 signaling (Koka et al. 2003; Prlic et al. 2003; Jamieson et al. 2004). In mice, the half‐life of NK cells after transfer into wild‐type mice was 7 days compared to only approximately 10 h in IL‐15R*α* ^−/−^mice (Koka et al. 2003). Similarly, a rapid reduction in NK cell count was also observed in cynomolgus monkeys after treatment with tofacitinib (CP‐690550) as described above (Conklyn et al. 2004; Borie et al. 2005).

The predicted PD parameters of Hu714MuXHu for NK cell count reduction in cynomolgus monkeys indicated a potent effect of this antibody, with an estimated mean EC_50_ value of approximately 0.1 *μ*g**·**mL^−1^ (%RSE 28.0), which is comparable to in vitro neutralization of 10 ng**·**mL^−1^ macaque IL‐10 by Hu714MuXHu in a PHA blast assay using cynomolgus macaque PBMCs (IC_50_ of 97 ng**·**mL^−1^; data on file at Amgen Inc.). *K*_in_, a measure of how fast NK cells are produced, was calculated to be 0.17 × 10^3^ cells·*μ*L^−1^·day^−1^, suggesting a rapid recovery of NK cells when IL‐15 signaling pathway is no longer inhibited. This matches the observation in PK and NK cell count profiles in Figures 2 and 3. However, due to the low EC_50_ value of 0.1 *μ*g**·**mL^−1^ and the long half‐life of Hu714MuXHu, a long duration of effect was to be expected. Indeed, in the 1‐month study, the NK cell number started to recover toward baseline values about 24 weeks after the last dose when the serum concentration fell below approximately 1 *μ*g**·**mL^−1^; approaching roughly 50% recovery at the end of the study, or 334 days after the first dose, when individual Hu714MuXHu concentrations were at or below the assay LLOQ of 0.1 *μ*g**·**mL^−1^. The PK/PD model‐estimated *K*_out_ of 0.305 day^−1^ can be translated into an elimination half‐life of 2.27 days for NK cells in monkeys. Using tofacitinib study data with NK cell count profiles in cynomolgus monkeys (Conklyn et al. 2004; Borie et al. 2005), and assuming an indirect response model for the NK cell depletion following the administration of tofacitinib, we used the initial slope of NK cell count‐time profiles to estimate *K*_out_ and subsequently the elimination half‐life of NK cells. The estimated values of 3–4.5 days in cynomolgus monkeys dosed with tofacitinib are generally consistent with our model‐estimated NK cell elimination half‐life of 2.27 days. However, caution should be exercised since we only used the initial slope for NK cell half‐life estimation and did not conduct PK/PD modeling analysis on the published tofacitinib data. In addition, these comparisons were conducted between different cynomolgus monkey studies where investigational products with different targets were administered (Hu714MuXHu vs. tofacitinib).

The described modeling approach could theoretically be used to select doses and dosing regimens for human clinical trials. Unexpectedly, administration of the anti‐IL‐15 antibody AMG 714 in humans did not result in a reduction in circulating NK cell counts despite AMG 714's ability to block human IL‐15 activity in in vitro assays and significantly higher exposure compared to the exposure achieved in cynomolgus monkeys as well as demonstration that the antibody in patient serum was still active (Lebrec et al. 2013). This observation triggered additional investigations which suggested that survival of NK cells is independent of IL–15 and that NK cell biology might differ significantly between species (i.e., humans and monkeys) (Lebrec et al. 2013). However, while IL‐15 might not be involved in the survival of NK cells in humans, a recent clinical study suggest that IL‐15 contributes to NK cell activation and proliferation in humans: administration of IL‐15 to cancer patients resulted in influx and hyperproliferation of NK cells in the peripheral blood (Conlon et al. 2015). Based on these data, it can be hypothesized that IL‐15 blockade might only affect NK cell counts under conditions where IL‐15 levels are elevated (e.g., in patients under certain inflammatory disease conditions) with possible effect of reducing NK cell numbers to levels observed in healthy volunteers.

In conclusion, after single and weekly intravenous or subcutaneous doses for up to 3 months in cynomolgus monkeys Hu714MuXHu displayed linear pharmacokinetics. The NK cell count reduction was assessed quantitatively using a model‐based approach. Although the reduction in NK cell counts was not observed in healthy volunteers after administration of AMG 714, the presented PK/PD modeling approach may be useful for potentially guiding future clinical trial dose selections in patients with inflammatory diseases where elevated IL‐15 concentrations have been observed.

## Disclosures

The authors are current or former employees and shareholders of Amgen Inc. except for S. T. Wolford, who is an employee of Covance Laboratories Inc.

## Author Contributions

Pan, Xiao, Horner, Lebrec, Butz, Kaliyaperumal, Buntich, Boren, Wolford, Tsuji, and Wienkers participated in the research design. Ortiz, Prokop, and Wolford conducted the experiments. Butz, Kaliyaperumal, Ortiz, and Prokop contributed new reagents or analytic tools. Pan, Li, Xiao, Horner, Lebrec, Cheah, and Köck performed data analysis. Pan, Li, Tsuji, and Köck wrote or contributed to the writing of the manuscript.
